# Supplementary material for: Cost-effectiveness of a home-based group educational programme on renal replacement therapies: a study protocol
Source: BMJ Open. 2019 Mar 23;9(3):e025684. doi: 10.1136/bmjopen-2018-025684 (PMC6475347; doi:10.1136/bmjopen-2018-025684)
Supplement: Author's manuscript [file bmjopen-2018-025684.draft_revisions.pdf]

BMJ Open

BMJ Open is committed to open peer review. As part of this commitment we make the peer review history of every article we publish publicly available.

When an article is published we post the peer reviewers' comments and the authors' responses online. We also post the versions of the paper that were used during peer review. These are the versions that the peer review comments apply to.

The versions of the paper that follow are the versions that were submitted during the peer review process. They are not the versions of record or the final published versions. They should not be cited or distributed as the published version of this manuscript.

BMJ Open is an open access journal and the full, final, typeset and author-corrected version of record of the manuscript is available on our site with no access controls, subscription charges or pay-per-view fees (http://bmjopen.bmj.com).

If you have any questions on BMJ Open's open peer review process please email info.bmjopen@bmj.com

BMJ Open

Cost-effectiveness of Educational Interventions Targeting Living Kidney Donation

Journal:	*BMJ Open*
Manuscript ID	bmjopen-2018-025684
Article Type:	Protocol
Date Submitted by the Author:	31-Jul-2018
Complete List of Authors:	Redeker, Steef; Erasmus Medical Center, Medical Psychology and Psychotherapy<br>Oppe, Mark ; EuroQol Research Foundation<br>Visser, Martijn; Erasmus MC, Psychiatry, Section Medical Psychology and Psychotherapy<br>Busschbach, Jan; Erasmus MC, Psychiatry, Section Medical Psychology and Psychotherapy<br>Weimar, Willem; Erasmus MC, Internal Medicine<br>Massey, Emma; Erasmus MC, Internal Medicine<br>Ismail, Sohal; Erasmus MC, Psychiatry, Section Medical Psychology and Psychotherapy
Keywords:	HEALTH ECONOMICS, End stage renal failure < NEPHROLOGY, Renal transplantation < NEPHROLOGY, Health policy < HEALTH SERVICES ADMINISTRATION & MANAGEMENT

SCHOLARONE™
Manuscripts

For peer review only

Cost-effectiveness of Educational Interventions Targeting Living Kidney Donation

Authors:

Steef Redeker, MSc[a]

Mark Oppe, PhD[b]

Martijn Visser, MSc[a]

Jan Busschbach, PhD[a]

Willem Weimar, PhD[c]

Emma K. Massey, PhD[c]

Sohal Ismail, PhD[a]

For the 'Nierteam aan Huis' consortium

Affiliations:

[a] Erasmus Medical Center, Section of Medical Psychology and Psychotherapy, Department of Psychiatry, Rotterdam, The Netherlands

[b] EuroQol Research Foundation, Rotterdam, The Netherlands.

[c] Erasmus Medical Center, Section of Transplantation, Department of Internal Medicine, The Netherlands

Contact information corresponding author

Steef Redeker

Erasmus MC, Psychiatry, Section Medical Psychology and Psychotherapy

Postal address: PO Box 2040, 3000 CA Rotterdam, The Netherlands

Work Phone: +31 10 704 38 05; Mobile Phone: +31 6 11 88 16 65

s.redeker@erasmusmc.nl

For peer review only - http://bmjopen.bmj.com/site/about/guidelines.xhtml

Participants of the Nierteam aan Huis consortium:

Bemelman, F., Pant, van der, K., Zwiers, N., Fleur, W. (AMC); Wetering, van de, J.,Weimar, W. Massey, E.K., Ismail, S.Y., Noord, van, M.A.A., Busschbach, J.J., Redeker, S., Berkel, van, I., Wageveld, K. (EMC); Clarisse, I., Hollander, D., Linde van de P., Dijk, van, C. (JBZ); Dorpel, van den, M. Rovers, E., Dongen, van, D., Kooij, van T., Marel, van der, T. (Maasstad Ziekenhuis); Brulez, H., Zwiers, L., Wisse, E. (OLVG West); Dooper, I., Hilbrands, L., Lobeek, M., Hopman, S. (Radboud UMC); Sanders, J., Berger, S., Waijer, J., Kisteman, M. (UMCG); Mahangoe, M., Dekker-Janssen, M., Hutten, F., Potgieter, J. (ZGT); Boonstra, C. (De Viersprong)

For peer review only

Trial Registration

Netherlands Trial Register, NTR6717

Keywords

Living donation, Kidney Transplantation, Cost-benefit analysis, kidney diseases, Health policy

Word count: 4155

For peer review only - http://bmjopen.bmj.com/site/about/guidelines.xhtml

Abstract

Introduction

Living donor kidney transplantation (LDKT) is the optimal treatment for most patients with end-stage renal disease (ESRD). However, there are numerous patients who cannot find a living kidney donor. Randomized controlled trials have shown that home-based education for patients with ESRD and their family/friends leads to four times more LDKTs. This educational intervention is currently being implemented in eight hospitals in the Netherlands. Supervision and quality assessment are being employed to map generalizability of the randomized trial while maintaining quality. There are a number of aims of this study: (1) to conduct a cost-effectiveness analysis of the educational program and its quality assurance system; (2) to investigate the relationship between the quality of the implementation of the intervention and the outcomes knowledge, communication, and LDKT activities; and (3) to investigate policy implications.

Methods and design

Patients with ESRD who do not have a living kidney donor are eligible to receive the home-based educational intervention. This is carried out by allied-health transplantation professionals across 8 hospitals in The Netherlands. The cost-effectiveness analysis will be conducted with a Markov model. Costs data will be obtained from the literature. We will obtain the quality of life data from the patients who are approached for the educational program. Questionnaires will be used to measure the outcomes of the program. Data on LDKT activities will be obtained from medical records up to 24 months after the education. Protocol adherence measures will be assessed by a third party by means of a telephone interview with the patients and the invitees.

Ethics and dissemination

Ethical approval is obtained through all participating hospitals. Results will be dissemnitated through peer-reviewed publications and scientific presentations. Results of the cost-effectiveness of the educational program will also be disseminated to the Dutch National Health Care Institute.

For peer review only

Strengths and limitations of this study

- Working with a strate-transition model involves a trade-off between feasibility and transparency of the model and the level of details of real life conditions.
- We do not have a control group in the implementation study, we will use the reported effectiveness of a randomized controlled trial.
- By making a dynamic state-transition model, we can model the prevalence and incidence of patients with end-stage renal disease, and consequently the capacity for the facility needs which has not been done before.
- We have a high quality data at our disposal.

For peer review only

Introduction

Living donor kidney transplantation (LDKT) is the optimal treatment for most patients with end-stage renal disease (ESRD) in terms of Quality Adjusted Life Years (QALYs), survival and cost-effectiveness [1]. However, there is a significant number of patients who cannot find a living kidney donor and many patients first undergo dialysis before transplantation with a living donor kidney. Interventions are needed to improve access to LDKT.

Research has shown that knowledge of renal replacement therapies (RRT) and communication between patients and their social circle play an important role in the access to LDKT [2]. Studies have shown that a home-based interventional program had positive effects for patients with ESRD [3-5]. In our transplant center in Rotterdam, the Netherlands, we have conducted two studies on this home-based educational approach: one randomized controlled trial (RCT) and one cross-over study. The RCT among 163 patients on dialysis showed significant increases in knowledge and communication regaring LDKT among the patients in the experimental arm who received the home-based education. Furthermore, there were significantly more actual LDKTs in the experimental group compared to the control group (17 vs 4, p=0.003) [3].

The cross-over trial was aimed at patients who had not previously undergone RRT and who were eligible for transplantation. In the first phase, the experimental arm received the home-based education while the control group waited. In the second phase, the control group also received the education. This study also showed that there was a statistically significant increase

For peer review only - http://bmjopen.bmj.com/site/about/guidelines.xhtml

in knowledge and communication regarding RRT among patients and invitees after receiving the home-based education. Of the 80 participants, 49 underwent RRT during the two year follow-up. Of these, 34 underwent a living donor kidney transplantation, of which 22 were pre-emptive [4].

Given these positive results, a home-based educational program for ESRD patients and their social network is currently being implemented in four regions in The Netherlands. Per region, a local hospital and a university transplant hospital are implementing the program. The local hospitals will target pre-dialysis patients, while the university hospitals will target both pre-dialysis and dialysis patients. The educators organize the intervention in such a way that they will do 'whatever it takes', in line with one of the basic principles of multisystem therapy (MST), to make this event as patient-tailored as possible. Supervision and quality assessment were employed to map the generalizability of previous research. The first aim is to evaluate the cost-effectiveness of the education to support continued implementation. In this article, we present the study protocol of a cost-effectiveness analysis of the educational program and its quality assurance system. The patient population, the standard care, the educations and the setting differ per hospital. Therefore, our second objective is to investigate the relationship between the quality of the implementation of the program, as measured by protocol adherence, and outcome. Outcome is defined in terms of knowledge, communication, and LDKT activities.

Hypotheses

Previous research has provided convincing evidence that transplantation cost less, gives a better survival and a higher quality of life compared to dialysis [6-8]. We therefore hypothesize that the relatively small incremental costs of the home-based educational program, through which the number of transplantations will increase, should be cost-effective. Since it is desirable that in the future all waiting list patients can benefit from the effects of the home-based intervention, this program should be part of standard care. Hence, a solid basis of the cost-effectiveness of that educational program is warranted.

The second hypothesis is that if healthcare providers show a high protocol adherence more positive intervention effects will occur. These effects include an increase of knowledge of renal disease and the treatment options, an increase in communication with family/friends about renal replacement therapies, an increase of living kidney donation activities, and an increase of

For peer review only - http://bmjopen.bmj.com/site/about/guidelines.xhtml

QALYs. If that relation between protocol adherence and effects is shown, a quality assurance system should be an inseparable part of the educational program.

The third hypothesis is that a full implementation of the educational program leads to policy implications regarding care for patients with ESRD. Full implementation may affect the need for dialysis centers and transplantation facilities. By modelling the prevalence we can estimate the need for allocating the health care budget. We aim to present the outcomes of the model in a budget impact analysis (BIA).

Thus, the main aim of this article is to present the implementation plan, to discuss the cost-effectiveness analysis and the quality assurance of the home-based educational program which currently being implemented in The Netherlands. Additionally, potential policy implications of our hypotheses are discussed in this article.

Methods and Design

Participants and procedure

The implementation study is being conducted in the following regions of the Netherlands: Rotterdam, Amsterdam, Nijmegen and Groningen. An estimate of the potential candidates for this implementation is about 50 patients per year per university center and 20 patients per local hospital. The implementation study will take two years.

The home-based educational program is currently being implemented in eight hospitals in the Netherlands; four university transplant hospitals and four local hospitals. Local hospitals were included to reach those patients who are yet to start renal replacement therapy and in this setting, this is the target population. In these hospitals there is a large dialysis unit but no transplants are conducted. The four university hospitals incorporate both a dialysis unit and a transplant center, therefore in this setting both pre-dialysis and dialysis patients are the target population.

If patients have not been able to find living donor candidates in their social network, they will be asked whether they and their social network wish to receive home-based education from health-care educators. The nephrologist explains to the patients that the educators will provide information about renal diseases and the different types of RRT. Furthermore, the educators can help to discuss the possibilities of living donation within the social network of the patient. If the patient consents to the intervention in consultation with his/her nephrologist, the health-care

For peer review only - http://bmjopen.bmj.com/site/about/guidelines.xhtml

educators contact the patient to make an appointment for the first home session. After completion of the program an evaluation consultation is planned with the nephrologist.

Patient and Public Involvement statement

Patients were involved in the design of the educational program. When designing the intervention we anchored the patient pariticipation in the project approach by relying on the results of focus groups in fifty patients from the intended target group. Their opinion was sought regarding two methods described in the literature of additional information/coaching: 1) an additional telephone consultation by the transplant doctor and 2) home education where family and friends are invited to receive knowledge about RRT. 88% of the participants favoured the home education over the telephone consultations [9]. Patients were not involved in the design of the cost-effectiveness study.

The intervention: home-based education

The intervention consists of two sessions at the patient's home. In the first session the goals of the educational program will be discussed and the home-based educational meeting will be prepared. The educators will make an inventory of individuals in the patient's social network using a socio-gram. This helps open the discussion on who to invite for the second session, the home-based educational meeting.

In the second session the education takes place. The educators organize this session in such a way that they will do 'whatever it takes', in line with one of the basic principles of multisystem therapy (MST), to make this event as patient-tailored as possible. The primary goal of this intervention is educational, therefore, it is not necessary that all the invitees are or become potential donors. A more detailed description can be found in a published protocol manuscript [10].

Measures

Knowledge and communication will be evaluated through questionnaires for all patients and for at least one relative/friend in attendance at the home-based educational session. The knowledge about renal disease and renal replacement therapies is measured through a validated knowledge questionnaire R3K-T. This 21-item knowledge questionnaire has been developed

For peer review only - http://bmjopen.bmj.com/site/about/guidelines.xhtml

specifically for kidney disease, and has good psychometric properties [11]. Answer categories are multiple choice and the number of correct answers are summed. The 3-item communication questionnaire can be answered on a scale from 1 (completely disagree) to 5 (completely agree). An example item is ‘I can talk about renal replacement therapies with my loved ones’. Finally we will assess patients’ and invitees’ attitude towards RRT through a 9-item questionnaire. This questionnaire can also be answered on a scale from 1 (completely disagree) to 5 (completely agree). An item example is ‘I am afraid donation will harm the health of the donor’. The administration of questionnaires will take place at two occasions 1) prior to the education either during an outpatient visit after signing the ‘informed consent’ or during the first session; and 2) shortly after the second session.

Protocol adherence measures: After every completed home intervention an independent telephone evaluation is conducted with the patient and a relative/friend who is involved in the education program, to measure the degree of protocol adherence of the educators. The independence is guaranteed through an independent party, specialized in treatment adherence measurement (www.Praktikon.nl). Protocol adherence in this implementation stands for the extent to which the different teams carry out the educational program as described in the protocol. Measurement is done with an adaptation of the ‘Treatment Adherence Measures’ (TAM) questionnaire [12]. TAM is scored on a 0-1 scale, where 1 stands for complete protocol adherence. The results of the TAM can not only be used for research purposes, but will also be used to give the educators feedback on the quality of their interventions during the implementation phase.

Cost-Effectiveness

Costs: The latest published research on costs of dialysis and transplantation in The Netherlands dates back from the late 1990s [6]. Currently, research on costs of dialysis and transplantation is in its final phase. We will use that forthcoming data (De Wit, personal communication). This cost data is of high quality, as it is based on the national database of insurance companies from 2014, which consists of records from 99% of all Dutch citizens. Costs calculations will include costs of dialysis modality, dialysis access, transplant procedure, other hospital costs, primary care costs, mental health care, medication outside the hospital, medical devices, health care abroad, transport, and other costs. Health care costs for transplantation,

For peer review only - http://bmjopen.bmj.com/site/about/guidelines.xhtml

include preparatory research, transplant operation, guidance, after care, donor expenses, dialysis procedure, other hospital costs, primary care, mental health care, medication outside hospital, medical devices, health care abroad, transportation and other costs. These include all the health care costs associated with RRT. Since it is preferred that cost-effectiveness analysis are conducted from a societal perspective, we also aim to calculate the productivity costs [13]. The costs of the home-based intervention and the quality assurance will be estimated on the basis of the current practice in the implementation.

Effects: In health economics the effects of interventions under evaluation are preferably expressed in QALYs [13]. Research on QALYs of different dialysis modalities are widespread [7, 14, 15], but instruments and patient background variables vary. Therefore, we are currently in the process of collecting quality of life data from both pre-dialysis patients and dialysis patients, through the EQ-5D-5L, the quality of life instrument recommended in the Dutch guidelines for health economics [16]. Moreover, we will also collect quality of life data of the patients who received the intervention.

Markov Model: To assess the cost-effectiveness of the implementation of the home-based educational program, we will build a 'Markov simulation model'. This model will assess the costs and effects of ESRD treatments as it simulates the course of treatment and disease of the patients. The model will have a similar structure as an earlier published model on this population [6], with some important updates and improvements. Unlike that Markov model, which used multivariate and univariate sensitivity analyses [6], our model will include probabilistic sensitivity analysis using Monte Carlo simulation. Consequently, uncertainty of all values are considered simultaneously, and the uncertainty in each parameter is assumed to possess a probability distribution [17]. By using probabilistic sensitivity analyses we follow current guidelines in health economics [16, 18, 19]. A Markov modelling technique is applicable because the decision problem involves risk that is continuous over time, the timing of events is important, and event may happen more than once [20]. Within a Markov simulation, the time horizon of the study is divided into a number of discrete time-periods, the so-called Markov cycles. A Markov process is based on the idea that patients are always in a certain disease state and that they can change between disease states once during each cycle. By assigning effects to each disease state and keeping track of the time patients remained in each disease state, long-term effects can be calculated.

For peer review only - http://bmjopen.bmj.com/site/about/guidelines.xhtml

Model description: A simplified graphic representation of the Markov model showing only the treatment categories, rather than all the individual Markov states is represented in figure 1. Patients continuously enter the model (inflow) at the start of the cycle and can start on Hemodialysis, Peritoneal dialysis, or Transplantation. From there they can move between these treatment modalities. Since the incidence of kidney failure is increasing, we will also model this in the cost-effectiveness analysis. This will be calculated through the database of *Nefrovisie*, a large national database with records of ESRD patients. As stated before, the model will include probabilistic analysis using Monte Carlo simulation. This means that not only mean numbers of patients per year per treatment modality will be estimated, but also the uncertainty surrounding those mean number of patients, 95% confidence intervals will be estimated with bootstrapping. This is a non-parametric technique in which the model parameters are estimated a large number of times. Consequently, all model parameters (e.g. the transition probabilities) will be included in the model as distributions rather than point estimates.

Markov States: The Markov states are based on the treatments currently available in The Netherlands. These are: Full Care Centre Hemodialysis, Limited Care Centre Hemodialysis, Home Hemodialysis, Continuous Ambulatory Peritoneal Dialysis, Continuous Cyclic Peritoneal Dialysis, Deceased Donor Kidney Transplantation, and Living Donor Kidney Transplantation. Since transition probabilities and costs may differ over time, i.e. a patient who is in his second year of hemodialysis has a different mortality chance than a patient who just started with hemodialysis, we will define separate Markov states for the 1st year of treatment and subsequent years of treatment with a specific modality. Incident patients that enter the model and prevalent patients that switch between treatment modalities are assigned to the 1st year Markov states, whereas patients that spend more than one year in any health state are transferred to the subsequent years of that same treatment modality.

Outcomes: The outcome of the implementation will be compared with the baseline situation; the situation before this program was implemented. A critical assumption will be the extrapolation of the effects after the 2 years.

Quality Assurance

This study has also implemented a central quality assurance. We hypothesize that the effectiveness is moderated by the protocol adherence of the team of healthcare professionals. In

For peer review only - http://bmjopen.bmj.com/site/about/guidelines.xhtml

the implementation study, it might be possible that there are differences in the way teams and centers adhere to the protocol. A part of the quality assurance is a training that all professionals that conduct the home interventions must take part in. During this training, issues are discussed such as: how to convey uniform and complete information to the patient, how to behave during the home visits, how to create an alignment of the goals of the home visit with the patients, how to assist the patient in inviting friends and relatives, how to deal with emotional moments, how to discuss delicate topic with respect to individual feelings and opinions and finally, how to ensure no detrimental psychosocial effects of the education occur for the patients and his/her family/friends. All these aspects can be executed in different ways by the educators. A supervisor will evaluate the home visits with each functional team separately every six weeks and discuss difficult cases. Furthermore, the supervisor will bring together all educators for a so-called intervision meeting every three months. These intervision sessions are meant to discuss the home visits with each other in order to learn from each other and to keep the procedures similar.

Discussion

We presented a protocol for assessing the cost-effectiveness of our home-based educational program and its generalizability. The implementation of the educational program might both benefit patients and society.

Cost-effectiveness: If indeed our hypotheses are confirmed, and the home-based educational program is cost-effective, then there are convincing arguments to make the program standard care in The Netherlands. Health insurers already expressed their interest in the program; this implementation study is supported by *Zorgverzekeraars Nederland (*Health insurers The Netherlands*)*, which is the ‘umbrella organization’ of all health insurers in The Netherlands. Additionally, the Dutch Kidney Foundation supports the program and contributed through three grants in the developmental phases of the home-based educational program. The Dutch Kidney Foundation is a non-profit organization which subsidizes research and innovation in nephrology and renal transplantation care. Indeed, the health insurance companies have good reason to be interested, as dialysis is costly. In The Netherlands, 1% of the total healthcare budget is spent on ESRD patients, who only constitute 0.0006% of the population [21, 22]. Furthermore, transplantation is associated with higher quality of life for ESRD patients compared to dialysis

For peer review only - http://bmjopen.bmj.com/site/about/guidelines.xhtml

treatment. It is therefore valuable, from both patient and societal perspective, to conduct a complete and extensive cost-effective analysis and consequently to follow up those results in terms of policy.

Quality System: Protocol adherence may be of importance to guarantee the effects of the home-based education. First, we expect a positive relation of adherence with outcome in terms of the quality of the decisions reached and the number of transplantations. Second, any problems or regrets of donors and/or patients can only be justified if the evidence based protocol was followed. The protocol has also been developed after thorough ethical consideration (23), which justified all characteristics of the program. It can therefore be argued that health insurance make reimbursement indispensable of the degree of protocol adherence of healthcare suppliers. Moreover, they should facilitate the quality assurance over the interventions and ensure that the quality of the interventions is independent of the healthcare suppliers.

*Limitations:*Investigating the (cost-)effectiveness of the home-based educational program has its limitations. In health economic modelling, there is always a trade-off between the feasibility and transparency of the cost-effectiveness model and the level of details of real life conditions as represented in the model. The more details, the more the model resembles real life, but the down side is that data should be available at that same level of detail and that the model becomes too complex in its feasibility. An example is that we assume that the mortality on dialysis is the same in the second and following years on dialysis. Hence, we know from literature that the mortality chance changes, but the data in later years is scarce and again the model would become more complex, as more 'tunnels states' have to be introduced. We expect that this trade-off will be most prominent in the transition changes between health states. We expect less obvious trade-offs for costs and utility assessment, as we have sufficient data for those variables.

Cooperation. This investigation is a cooperation between many parties, who all have expressed their support. Obviously, it is possible that this support can be withdrawn for several reasons. For instance, we depend on data from a large national database with records of patient with ESRD (*Nefrovisie*). Hence, much efforts are and will be put in preserving relationships and communication in order to maximize fair successful implementation chances for the program.

Ethical considerations. Another challenge that we face, is the ethical consideration of promoting living kidney donation through a home-based intervention. Previous studies on the

For peer review only - http://bmjopen.bmj.com/site/about/guidelines.xhtml

ethics of this argued that such promotion is justified, only when the conditions are met, such as 1) participation must be completely voluntary throughout the intervention, 2) no undue pressure should be put on the participants, 3) the education is neutral and non-directive and 4) the purpose and the procedure should be clear to all participants [23, 24]. That does not mean there are no negative consequence whatsoever, but it does mean that the positive outcomes outweigh the negative. It could well be that a patient and/or a donor may regret the decision to have undertaken a transplantation with a kidney of a living donor, and that the donor and/or patient, in hindsight, may have felt undue pressure to donate a kidney. Adherence to the protocol will minimize these potential negative effects. However, it is possible that regret or pressure could lead to negative publicity for the program. That is a risk since such negative publicity could impede the chances of implementing the program as standard care [25]. Especially considering that deceased organ donation is currently subject to controversy in The Netherlands since the government and parliament are currently debating a new donor law. This proposed law is an opt-out system entailing a positive 'no-objection' deceased donor organ donor registration as a default for all Dutch citizens [26]. Given this potential harm due to negative publicity it is crucial to have 1) a protocol which is justifiable from a medical ethical perspective, 2) widespread support from the various organizations involved and 3) a high quality in terms of protocol adherence and trained adequate educators. If these conditions are met, the quality of the process then justify its outcome, which is a subtle trade-off between positive and negative outcomes.

Implementation. Another challenge is the generalizability of the results of the previous effect study on home-based interventions done in Rotterdam. The randomized controlled trial in the Rotterdam transplant area has shown that the home-based educational program leads up to four times more LDKTs. The trial took place at the academic transplant center (Erasmus Medical Center in Rotterdam) where extensive efforts were already undertaken as part of the standard care to promote LDKTs [3, 27]. It is possible that in other transplant areas in The Netherlands, with less experience regarding promoting LDKT, effectiveness in terms of amount of LDKTs may differ. On one hand this more cautious attitude may lead to lower results than in the Rotterdam transplant area. Hence, organizational conditions within those transplant centers may not optimally facilitate the favorable results of the interventions. On the other hand, if the number of LDKT was lower than in the Rotterdam area, and the uptake of the intervention is

For peer review only - http://bmjopen.bmj.com/site/about/guidelines.xhtml

high, the effect could even be higher than in the Rotterdam area. This is due to a higher effect potential in those centers where living donation was not promoted as actively.

Learning curve. As with all new programs, educators will inherently experience a learning curve during the first part of the implementation, which could influence the effectiveness of the program. For instance, the goal of 50 patients per year per academic transplant center and the goal of 20 patients per regional hospital may not be reached. Regular supervision, (on the job) training and peer-to-peer coaching may help to overcome this, but a learning curve is unavoidable.

Policy implications: One of the main pillars of an efficient health care system is the ability to provide effective care to patients when needed. It is therefore necessary to have information on the effectiveness of the interventions and their cost to convince policy makers to reimburse the treatment. If the analysis confirms the effectiveness as well as the cost-effectiveness of the home-based educational program, we recommend that this intervention should be part of standard-care.

If the home-based educational interventions would become standard-care, this could have several implications. First, it can be expected that patients who are unable to find a living donor will nevertheless profit from an increase number of living kidney donations, as the demand for deceased donor kidneys drops. In other words, the increase in living donation will further lower the waiting list for deceased donor donation as well and thereby increase the chance of a deceased donor donation for those patients without a living donor increases.

Second, the composition of the population of patients with ESRD may change. For instance, it can be expected that the proportion of patients on dialysis will drop and patients with a life sustaining transplanted kidney will increase. This might have an influence on the demand for dialysis centers and the need for transplantation facilities. We, therefore, aim to incorporate this in a so-called dynamic model to estimate the prevalence over time. When modelling the prevalence, we could make estimates of the need for dialysis centers and transplantations facilities. However, modelling the facility is surrounded by uncertainty. For instance, dialysis centers may have financial incentives to fulfill dialysis capacity. If this would be the case, there will be no monetary benefits for society by increasing the transplants facilities if the proportion of dialysis capacity remains the same. This would mean that there will only be an increase in the average quality of life of ESRD-patients.

For peer review only - http://bmjopen.bmj.com/site/about/guidelines.xhtml

Finally, a cost-effectiveness analysis only might not be sufficient to set policy change in motion. Therefore, we anticipate that a policy recommendation accompanied with a BIA will also be required. A BIA addresses the expected changes in the expenditure of a health care system after the adoption of a new intervention. It can also be used for budget and resource planning [28, 29].

Conclusion: If our hypotheses are confirmed, we hope by presenting an extensive cost-effectiveness analysis, a BIA, and a policy recommendation that policy change will be set in motion, which again would benefit both ESRD-patients and society.

For peer review only

Declarations

Abbreviations

BIA: Budget Impact Analysis; ESRD: End-Stage Renal Disease; LDKT: Living Donor Kidney Transplantation; RCT: Randomized Controlled Trial; RRT: Renal Replacement Therapies; QALY: Quality Adjusted Life Years; TAM; Treatment Adherence Measures.

Acknowledgements

All authors express their gratitude to the participating hospitals, the educators and their supervisors.

Funding

The research is funded by the Dutch Kidney Foundation. The implementation is supported by Dutch Health Insurers.

Availability of data and materials

Not applicable.

Author contributions

SR drafted the manuscript. MO, MV, JB, WW, EM, SI provided significant critical intellectual contributions. All authors reviewed and approved the final version of this manuscript and agrees to be accountable for all aspects of this work.

Consent for publication

Not applicable.

Competing interests

All authors have no competing interests to report.

For peer review only - http://bmjopen.bmj.com/site/about/guidelines.xhtml

References

1. Mahillo B, Carmona M, Alvarez M, White S, Noel L, Matesanz R: **2009 global data in organ donation and transplantation: activities, laws, and organization**. *Transplantation* 2011, **92**(10):1069-1074.
2. Kranenburg LW, Zuidema WC, Weimar W, Hilhorst MT, IJzermans JN, Passchier J, Busschbach JJ: **Psychological barriers for living kidney donation: how to inform the potential donors**. *Transplantation* 2007, **84**:965-971.
3. Ismail SY, Luchtenburg AE, Timman R, Zuidema WC, Boonstra C, Weimar W, Busschbach JJ, Massey EK: **Home-based family intervention increases knowledge, communication and living donation rates: a randomized controlled trial**. *Am J Transplant* 2014, **14**(8):1862-1869.
4. Massey EK, Smak Gregoor PJH, Nette RW, van den Dorpel MA, van Kooij A, Zietse R, Zuidema WC, Timman R, Busschbach JJ, Weimar W: **Early home-based group education to support informed decision-making among patients with end-stage renal disease: a multi-centre randomized controlled trial**. *Nephrol Dial Transplant* 2016, **31**:823-830.
5. Rodrigue JR, Cornell DL, Lin JK, Kaplan B, Howard RJ: **Increasing live donor kidney transplantation: a randomized controlled trial of a home-based educational intervention**. *Am J Transplant* 2007, **7**(2):394-401.
6. de Wit GA, H. RP, de Charro FT: **Eonomic evaluation of end stage renal disease treatment**. *Health policy* 1998, **44**(3):215-232.
7. Wyld M, Morton RL, Hayen A, Howard K, Webster AC: **A systematic review and meta-analysis of utility-based quality of life in chronic kidney disease treatments**. *PLoS Med* 2012, **9**(9):e1001307.
8. Liem YS, Bosch JL, Hunink MG: **Preference-based quality of life of patients on renal replacement therapy: a systematic review and meta-analysis**. *Value Health* 2008, **11**(4):733-741.
9. Ismail SY, Claassens L, Luchtenburg AE, Roodnat JI, Zuidema WC, Weimar W, Busschbach JJ, Massey EK: **Living donor kidney transplantation among ethnic minorities in the Netherlands: a model for breaking the hurdles**. *Patient Educ Couns* 2013, **90**(1):118-124.
10. Ismail SY, Luchtenburg AE, Zuidema WC, Boonstra C, Weimar W, Massey EK, Busschbach JJ: **Multisystemic engagement & nephrology based educational intervention: a randomized controlled trial protocol on the kidney team at home-study**. *BMC Nephrology* 2012, **13**:62-70.
11. Ismail SY, Timmerman L, Timman R, Luchtenburg AE, Smak Gregoor PJH, Nette RW, van den Dorpel MA, Zuidema WC, Weimar W, Massey EK *et al*: **A psychometric analysis of Rotterdam Renal Replacement Knowledge-Test (R3K-T) using item response theory**. *Transplant International* 2013, **26**:1164-1172.

For peer review only - http://bmjopen.bmj.com/site/about/guidelines.xhtml

12. Chapman JE, Sheidow AJ, Henggeler SW, Halliday-Boykins C, Cunningham PB: **Developing a Measure of Therapist Adherence to Contingency Management: An Application of the Many-Facet Rasch Model**. *J Child Adolesc Subst Abuse* 2008, **17**(3):47-68.
13. Drummond MF, Sculpher MJ, Claxton K, Stoddart GL, Torrance GW: **Methods for the Economic Evaluation of Health Care Programmes**. Oxford: Oxford University; 2015.
14. Harris A, Cooper BA, Li JJ, Bulfone L, Branley P, Collins JF, Craig JC, Fraenkel MB, Johnson DW, Kesselhut J *et al*: **Cost-effectiveness of initiating dialysis early: a randomized controlled trial**. *Am J Kidney Dis* 2011, **57**(5):707-715.
15. Wu AW, Fink NE, Marsh-Manzi JV, Meyer KB, Finkelstein FO, Chapman MM, Powe NR: **Changes in quality of life during hemodialysis and peritoneal dialysis treatment: generic and disease specific measures**. *J Am Soc Nephrol* 2004, **15**(3):743-753.
16. Nederland Z: **Richtlijn voor het uitvoeren van economische evaluaties in de gezondheidszorg**. In. Diemen: Zorginstituut Nederland; 2016.
17. Doubilet P, Begg CB, Weinstein MC, Braun P, J. MB: **Probabilistic Sensitivity Analysis Using Monte Carlo Simulation**. *Med Decis Making* 1985, **5**(2):157-177.
18. Briggs AH: **Handling uncertainty in cost-effectiveness models**. *Pharmacoeconomics* 2000, **17**(5):479-500.
19. Briggs AH, Goeree R, Blackhouse G, O'Brien BJ: **Probabilistic analysis of cost-effectiveness models: choosing between treatment strategies for gastroesophage reflux disease**. *Med Decis Making* 2002, **22**:290-308.
20. Sonnenberg FA, Beck JR: **Markov Models in Medical Decision Making: A Pratical Guide**. *Med Decis Making* 1993, **13**:322-338.
21. **Ranglijst ziekten op basis van zorgkosten** [https://www.volksgezondheidenzorg.info/ranglijst/ranglijst-ziekten-op-basis-van-zorgkosten]
22. **Feiten en cijfers over behandeling nierfalen** [https://www.nierstichting.nl/leven-met-een-nierziekte/feiten-en-cijfers/]
23. Hilhorst MT, Kranenburg LW, Busschbach JJ: **Should health care professionals encourage living kidney donation**. *Medicine, Health Care and Philosophy* 2006, **10**:81-90.
24. Massey EK, Hilhorst MT, Nette RW, Smak Gregoor PJH, van den Dorpel MA, van Kooij A, Zuidema WC, Zietse R, Busschbach JJ, Weimar W: **Justification for a home-based education programme for kidney patients and their social network prior to initiation of renal replacement therapy**. *J Med Ethics* 2011, **37**:677-681.
25. Birkland TA: **Focusing Events, Mobilization, and Agenda Setting**. *Journal of Public Policy* 1998, **18**(1):53-74.

For peer review only - http://bmjopen.bmj.com/site/about/guidelines.xhtml

26. Eleftheriou-Smith L: **Organ Donation set to become automatic for Dutch people unless they opt out**. In: *Independent.* 2016.
27. Roodnat JI, Kal-van Gestel JA, Zuidema W, van Noord MA, van de Wetering J, JN IJ, Weimar W: **Successful expansion of the living donor pool by alternative living donation programs**. *Am J Transplant* 2009, **9**(9):2150-2156.
28. Niezen MG, de Bont A, Busschbach JJ, Cohen JP, Stolk EA: **Finding legitimacy for the role of budget impact in drug reimbursement decisions**. *Int J Technol Assess Health Care* 2009, **25**(1):49-55.
29. Sullivan SD, Mauskopf JA, Augustovski F, Caro JJ, Lee KM, Minchin M, Orlewska E, Penna P, Rodriguez Barrios J-M, Wen-Yi S: **Budget Impact Analysis - Principles of Good Practice: Report of the ISPOR 2012 Buget Impact Analysis Good Practice II Task Force**. *Value in Health* 2014, **17**:5-14.

Figure legends

Figure 1: A simplified graphic representation of the Markov Model with the different health states and the transition possibilities between the health states.

For peer review only

For peer review only - http://bmjopen.bmj.com/site/about/guidelines.xhtml

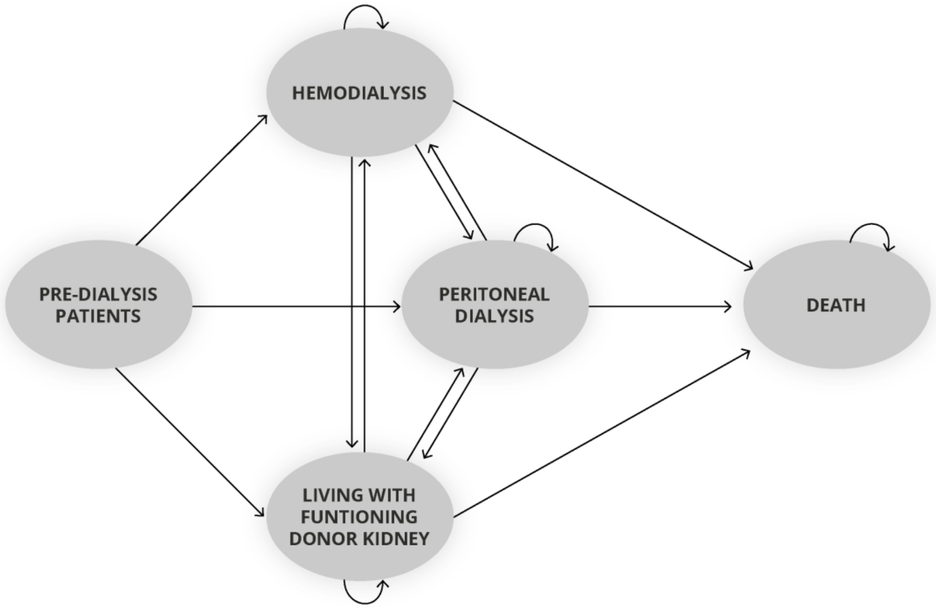

A simplified graphic representation of the Markov Model with the different health states and the transition possibilities between the health states.

234x151mm (300 x 300 DPI)

Cost-effectiveness of a home-based group educational programme on renal replacement therapies: a Study Protocol

Journal:	*BMJ Open*
Manuscript ID	bmjopen-2018-025684.R1
Article Type:	Protocol
Date Submitted by the Author:	06-Nov-2018

Complete List of Authors:	Redeker, Steef; Erasmus MC, Psychiatry, Section Medical Psychology and Psychotherapy<br>Oppe, Mark ; EuroQol Research Foundation<br>Visser, Martijn; Erasmus MC, Psychiatry, Section Medical Psychology and Psychotherapy<br>Busschbach, Jan; Erasmus MC, Psychiatry, Section Medical Psychology and Psychotherapy<br>Weimar, Willem; Erasmus MC, Internal Medicine<br>Massey, Emma; Erasmus MC, Internal Medicine<br>Ismail, Sohal; Erasmus MC, Psychiatry, Section Medical Psychology and Psychotherapy

**Primary Subject Heading**:	Health economics
Secondary Subject Heading:	Health policy
Keywords:	HEALTH ECONOMICS, End stage renal failure < NEPHROLOGY, Renal transplantation < NEPHROLOGY, Health policy < HEALTH SERVICES ADMINISTRATION & MANAGEMENT

SCHOLARONE™ Manuscripts

Cost-effectiveness of a home-based group educational programme on renal replacement therapies: a Study Protocol

Authors:

Steef Redeker, MSc[a]

Mark Oppe, PhD[b]

Martijn Visser, MSc[a]

Jan Busschbach, PhD[a]

Willem Weimar, PhD[c]

Emma K. Massey, PhD[c]

Sohal Ismail, PhD[a]

For the 'Nierteam aan Huis' consortium

Affiliations:

[a] Erasmus MC, University Medical Center Rotterdam, Department of Psychiatry, The Netherlands

[b] EuroQol Research Foundation, Rotterdam, The Netherlands.

[c] Erasmus MC, University Medical Center Rotterdam, Department of Internal Medicine, The Netherlands

Contact information corresponding author

Steef Redeker

Erasmus MC, Psychiatry, Section Medical Psychology and Psychotherapy

Postal address: PO Box 2040, 3000 CA Rotterdam, The Netherlands

Work Phone: +31 10 704 38 05; Mobile Phone: +31 6 11 88 16 65

s.redeker@erasmusmc.nl

For peer review only - http://bmjopen.bmj.com/site/about/guidelines.xhtml

Participants of the Nierteam aan Huis consortium:

Bemelman, F., Pant, van der, K., Zwiers, N., Van Milgen-Adriaens, M. (AMC); Wetering, van de, J.,Weimar, W. Massey, E.K., Ismail, S.Y., Noord, van, M.A.A., Busschbach, J.J., Redeker, S.,Jansen, D.., Wageveld, K. (EMC); Clarisse, I., Hollander, D., Linde van de P., Dijk, van, C. (JBZ); Dorpel, van den, M. Rovers, E., Dongen, van, D., Kooij, van T., Marel, van der, T. (Maasstad Ziekenhuis); Brulez, H., Zwiers, L., Wisse, E. (OLVG West); Dooper, I., Hilbrands, L., Lobeek, M., Hopman, S. (Radboud UMC); Sanders, J., Berger, S., Waijer, J., Kisteman, M., Van der Laan, J. (UMCG); Mahangoe, M., Dekker-Janssen, M., Hutten, F., Potgieter, J. (ZGT); Boonstra, C. (De Viersprong)

For peer review only

Trial Registration

Netherlands Trial Register, NTR6717

Keywords

Living donation, Kidney Transplantation, Cost-benefit analysis, kidney diseases, Health policy

Word count: 4155

For peer review only - http://bmjopen.bmj.com/site/about/guidelines.xhtml

Abstract

Introduction

Living donor kidney transplantation (LDKT) is the optimal treatment for most patients with end-stage renal disease (ESRD). However, there are numerous patients who cannot find a living kidney donor. Randomized controlled trials have shown that home-based education for patients with ESRD and their family/friends leads to four times more LDKTs. This educational intervention is currently being implemented in eight hospitals in the Netherlands. Supervision and quality assessment are being employed to maintain the quality of the intervention. In this study we aim to: (1) conduct a cost-effectiveness analysis of the educational programme and its quality assurance system; (2) investigate the relationship between the quality of the implementation of the intervention and the outcomes knowledge, communication, and LDKT activities; and (3) investigate policy implications.

Methods and design

Patients with ESRD who do not have a living kidney donor are eligible to receive the home-based educational intervention. This is carried out by allied-health transplantation professionals and psychologists across 8 hospitals in The Netherlands. The cost-effectiveness analysis will be conducted with a Markov model. Cost data will be obtained from the literature. We will obtain the quality of life data from the patients who participate in the educational programme. Questionnaires on knowledge and communication will be used to measure the outcomes of the programme. Data on LDKT activities will be obtained from medical records up to 24 months after the education. A protocol adherence measure will be assessed by a third party by means of a telephone interview with the patients and the invitees.

Ethics and dissemination

Ethical approval was obtained through all participating hospitals. Results will be dissemnitated through peer-reviewed publications and scientific presentations. Results of the cost-effectiveness of the educational programme will also be disseminated to the Dutch National Health Care Institute.

For peer review only

For peer review only - http://bmjopen.bmj.com/site/about/guidelines.xhtml

Strengths and limitations of this study

- Working with a strate-transition model involves a trade-off between feasibility and transparency of the model and the level of details of real life conditions.
- We do not have a control group in the implementation study, we will use the reported effectiveness of a randomized controlled trial conducted previously.
- By making a dynamic state-transition model, we can model the prevalence and incidence of patients with end-stage renal disease, and consequently the capacity for the facility needs which has not been done before.
- We have high quality data at our disposal.

Introduction

Living donor kidney transplantation (LDKT) is the optimal treatment for most patients with end-stage renal disease (ESRD) in terms of Quality Adjusted Life Years (QALYs), survival and cost-effectiveness [1]. However, there is a significant number of patients who cannot find a living kidney donor and many patients first undergo dialysis before transplantation with a living donor kidney. Interventions are needed to improve access to LDKT.

Research has shown that knowledge of renal replacement therapies (RRT) and communication between patients and their social circle play an important role in the access to LDKT [2]. Studies have shown that a home-based interventional programme had positive effects for patients with ESRD [3-5]. In our transplant center in Rotterdam, the Netherlands, we have conducted two studies on this home-based educational approach: one randomized controlled trial (RCT) and one cross-over study. The RCT among 163 patients on dialysis showed significant increases in knowledge and communication about LDKT among the patients in the experimental arm who received the home-based education compared to the standard care control arm. Furthermore, there were significantly more actual LDKTs in the experimental group compared to the control group (17 vs 4, p=0.003) [3].

The cross-over trial was aimed at patients who had not previously undergone RRT and who were eligible for transplantation. In the first phase, the experimental arm received the home-based education while the control group waited. In the second phase, the control group also received the education. This study also showed that there was a statistically significant increase

For peer review only - http://bmjopen.bmj.com/site/about/guidelines.xhtml

in knowledge and communication regarding RRT among patients and invitees after receiving the home-based education. Of the 80 participants, 49 underwent RRT during the two year follow-up. Of these, 34 underwent a living donor kidney transplantation, of which 22 were pre-emptive [4].

Given these positive results, a home-based educational programme for ESRD patients and their social network is currently being implemented in four regions in The Netherlands. Per region, a regional hospital and a university transplant hospital are implementing the programme. The regional hospitals will target patients who are yet to start renal replacement therapy, while the university hospitals will target both these patients and dialysis patients. The educators organize the intervention in such a way that they will do 'whatever it takes', in line with one of the basic principles of multisystem therapy (MST), to make this event as patient-tailored as possible [6]. Supervision and quality assessment are being employed to mantain the quality of the intervention. The first aim of this study is to evaluate the cost-effectiveness of the education to support continued implementation. In this article, we present the study protocol of a cost-effectiveness analysis of the educational programme and its quality assurance system. The patient population, the standard care, the quality of the educations and the setting differ per hospital. Therefore, our second objective is to investigate the relationship between the quality of the implementation of the programme, as measured by protocol adherence, and outcome. Outcome is defined in terms of knowledge, communication, and LDKT activities.

Hypotheses

Previous research has provided convincing evidence that transplantation cost less, gives a better survival and a higher quality of life compared to dialysis [7-9]. We therefore hypothesize that the relatively small incremental costs of the home-based educational programme, should be cost-effective. Since it is desirable that in the future all waiting list patients can benefit from the effects of the home-based intervention, this programme should be part of standard care. Hence, a solid basis of the cost-effectiveness of that educational programme is warranted.

The second hypothesis is that higher protocol adherence among healthcare providers will be associated with more positive effects of the educational interventions. These effects include an increase in knowledge of renal disease and the treatment options, an increase in communication with family/friends about renal replacement therapies, an increase in living kidney donation activities, and an increase in QALYs. If a relationship between protocol

For peer review only - http://bmjopen.bmj.com/site/about/guidelines.xhtml

adherence and effects is shown, a quality assurance system should be an inseparable part of the educational programme.

The third hypothesis is that a full implementation of the educational programme leads to policy implications regarding care for patients with ESRD. Full implementation may affect the need for dialysis centers and transplantation facilities. By modelling the prevalence we can estimate the need for allocating the health care budget. We aim to present the outcomes of the model in a budget impact analysis (BIA).

Thus, the main aim of this article is to discuss the protocol of the cost-effectiveness study of the home-based educational programme and of the quality assurance programme which currently being implemented in The Netherlands. Additionally, potential policy implications of our hypotheses are discussed in this article.

Methods and Design

Participants and procedure

The implementation study is being conducted in the following regions of the Netherlands: Rotterdam, Amsterdam, Nijmegen and Groningen.

The home-based educational programme is currently being implemented in eight hospitals in the Netherlands; four university transplant hospitals and four regional hospitals. Regional hospitals were included to reach those patients who are yet to start renal replacement therapy and in this setting, this is the target population. In these hospitals there is a large dialysis unit but no transplants are conducted. For these hospitals, the inclusion criteria are: ≥18 years of age, are eligible for transplantation, and primary RRT required within the coming 12 months. In the regional hospitals allied health professionals carry out the intervention. The four university hospitals incorporate both a dialysis unit and a transplant center, therefore in this setting both patients who are yet to start renal replacement therapy and dialysis patients are the target population. Eligible patients for these hospitals are required to be ≥18 years, currently undergoing RRT or required within the coming 12 months and eligible for transplantation. In the university hospitals, allied health professionals are accompanied by psychologists to carry out the intervention. The distinction between the university hospitals and the regional hospitals is in line with the protocols of the aforementioned Cross-over study and the RCT.An estimate of the

For peer review only - http://bmjopen.bmj.com/site/about/guidelines.xhtml

potential candidates for this implementation is about 50 patients per year per university center and 20 patients per regional hospital. The implementation study will take two years.

If patients have not been able to find living donor candidates in their social network, they will be asked whether they and their social network wish to receive home-based education from health-care educators. The nephrologist explains to the patients that the educators will provide information about renal diseases, the different types of RRT and their impact on quality of life. Furthermore, the educators can help to discuss the possibilities of living donation within the social network of the patient. If the patient consents to the intervention in consultation with his/her nephrologist, the health-care educators contact the patient to make an appointment for the first home session. Patients are supported in inviting their family/friends to the second educational session. After completion of the programme an evaluation consultation is planned with the nephrologist. The number of patients who do not consent to participate in the study is recorded, as well as the reason for nonparticipation.

Patient and Public Involvement statement

Patients were involved in the design of the educational programme. When designing the intervention we anchored the patient pariticipation in the project approach by relying on the results of focus groups among fifty patients from the intended target group. Their opinion was sought regarding two methods described in the literature of additional information/coaching: 1) an additional telephone consultation by the transplant doctor [10] and 2) home education where family and friends are invited to receive knowledge about RRT. Eighty-eight percent of the participants favoured the home-based education over the telephone consultations [11]. Additionally, a patient panel and organizations were involved in the development of the educational programme protocol and materials. Patients were not involved in the design of the cost-effectiveness study.

The intervention: home-based education

The intervention consists of two sessions at the patient's home. The intervention is carried out by allied-health transplantation professionals and psychologists. In the first session the goals of the educational programme are discussed and the home-based educational meeting will be prepared. The educators will make an inventory of individuals in the patient's social

For peer review only - http://bmjopen.bmj.com/site/about/guidelines.xhtml

network using a socio-gram. This helps open the discussion on who to invite for the second session, the home-based educational meeting. The need for an independent translator is also dicussed.

In the second session the education takes place. The educators organize this session in such a way that they will do 'whatever it takes', in line with one of the basic principles of multisystem therapy (MST), to make this event as patient-tailored as possible [6]. The primary goal of this intervention is educational, therefore, it is not necessary that all the invitees are or become potential donors. The intervention is based on the previous RCT and Cross-Over studies [3, 4]. A more detailed description can be found in a published protocol manuscript [12].

Measures

Knowledge and communication are evaluated through questionnaires among all patients and for at least one relative/friend in attendance at the home-based educational session. The knowledge about renal disease and renal replacement therapies is measured through a validated knowledge questionnaire R3K-T. This 21-item knowledge questionnaire has been developed specifically for kidney disease, and has good psychometric properties [13]. Answer categories are multiple choice and the number of correct answers are summed. The 3-item communication questionnaire can be answered on a scale from 1 (completely disagree) to 5 (completely agree). An example item is 'I can talk about renal replacement therapies with my loved ones'. Finally we assess patients' and invitees' attitude towards RRT through a 9-item questionnaire. This questionnaire can also be answered on a scale from 1 (completely disagree) to 5 (completely agree). An item example is 'I am afraid donation will harm the health of the donor'. The administration of questionnaires will take place at two occasions 1) prior to the education either during an outpatient visit after signing the 'informed consent form' or during the first session; and 2) shortly after the second session.

Protocol adherence measures: After every completed home intervention an independent telephone evaluation is conducted with the patient and a relative/friend who attended the education programme, to measure the degree of protocol adherence of the educators. The independence is guaranteed through an independent party, specialized in treatment adherence measurement (www.Praktikon.nl). Protocol adherence in this implementation is defined as the extent to which the different teams carry out the educational programme as described in the

For peer review only - http://bmjopen.bmj.com/site/about/guidelines.xhtml

protocol. Measurement is done with an adaptation of the 'Treatment Adherence Measures' (TAM) questionnaire [14]. TAM is scored on a 0-1 scale, where 1 stands for complete protocol adherence. The results of the TAM can not only be used for research purposes, but also to give the educators feedback on the quality of their interventions during the implementation phase.

Cost-Effectiveness

Costs: The latest published research on costs of dialysis and transplantation in The Netherlands dates back from the late 1990s [7]. Currently, research on costs of dialysis and transplantation is in its final phase. We will use this forthcoming data (De Wit, personal communication). This cost data is of high quality, as it is based on the national database of insurance companies from 2014, which consists of records from 99% of all Dutch citizens. Costs calculations will include costs of dialysis modality, dialysis access, transplant procedure, other hospital costs, primary care costs, mental health care, medication outside the hospital, medical devices, health care abroad, transport, and other costs. Health care costs for transplantation, include preparatory research, transplant operation, guidance, after care, donor expenses, dialysis procedure, other hospital costs, primary care, mental health care, medication outside hospital, medical devices, health care abroad, transportation and other costs. These include all the health care costs associated with RRT. Since it is recommended that cost-effectiveness analysis are conducted from a societal perspective, we also aim to calculate the productivity costs [15]. Productivity costs will be estimated with the friction-cost method, as recommended in the Dutch guidelines for economic evaluation [16]. Besides that the work situation of patients participating in the study is recorded, some research has been done regarding labor participation of patients with ESRD [17, 18]. The costs of the home-based intervention and the quality assurance will be estimated on the basis of the current practice in the implementation. Informal care costs will be estimated from the literature [19]. The costs of the intervention are recorded per center.

Effects: In health economics the effects of interventions under evaluation are preferably expressed in QALYs [15]. Research on QALYs of different dialysis modalities are widespread [8, 20, 21], but instruments and patient background variables vary. Therefore, we are currently in the process of collecting quality of life data from both patients who are yet to start renal replacement therapy and dialysis patients prior to the intervention, through the EQ-5D-5L, the quality of life instrument recommended in the Dutch guidelines for health economics [16]. We

For peer review only - http://bmjopen.bmj.com/site/about/guidelines.xhtml

also collect quality of life data from patients after the intervention. The educators administer the EQ-5D-5L questionnaire to patients at baseline, 6, 12 and 24 months after the intervention by telephone.

Markov Model: To assess the cost-effectiveness of the implementation of the home-based educational programme, we will build a 'Markov simulation model'. This model will assess the costs and effects of ESRD treatments as it simulates the course of treatment and disease of the patients. The model will have a similar structure as a previously published model on this population [7], with some important updates and improvements. Unlike that Markov model, which used multivariate and univariate sensitivity analyses [7], our model will include probabilistic sensitivity analysis using Monte Carlo simulation. Consequently, uncertainty of all values are considered simultaneously, and the uncertainty in each parameter is assumed to possess a probability distribution [22]. The model will run 10,000 simulations in Microsoft Excel, version 2010. By using probabilistic sensitivity analyses we follow current guidelines in health economics [16, 23, 24]. A Markov modelling technique is applicable because the decision problem involves risk that is continuous over time, the timing of events is important, and events may happen more than once [25]. Within a Markov simulation, the time horizon of the study is divided into a number of discrete time-periods, the so-called Markov cycles. A Markov process is based on the idea that patients are always in a certain disease state and that they can change between disease states once during each cycle. By assigning effects to each disease state and keeping track of the time patients remained in each disease state, long-term effects can be calculated. For this cost-effectiveness analysis the effects and cost per health state do not change because of the intervention. We expect that there will be more LDKTs because of the intervention. Therefore, besides the costs of the programme, only the transition probabilities will change because of the intervention and will be the only difference between the baseline and the post-implementation situation. Table 1 shows an overview of the parameters used and the source.

For peer review only

For peer review only - http://bmjopen.bmj.com/site/about/guidelines.xhtml

Table 1: overview of parameters and sources in the Markov Model:

Parameter	Sources
**Costs**<br>1. Medical Costs<br>2. Intervention Costs<br>3. Costs of Productivity Losses<br>4. Informal Care Costs	1. Costs study of RRT in The Netherlands by de Wit et al. (Forthcoming)<br>2. Are recorded in the current implementation study<br>3. Work situation of patients is recorded in the implementation study and use of (Dutch) literature [16, 17]<br>4. Will be estimated from existing literature [18].
**Effects (QALYs)**	EQ-5D-5L are conducted prior at the intervention and 6, 12 and 24 months after the intervention.
**Transition probabilities**<br>1. Between treatment modalities<br>2. Mortality rate	Estimated from the database of Nefrovisie
**Incidence rates**	Estimated from the database of Nefrovisie
**Effect size of the intervention**	Used from previous studies [3, 4]

Model description: A simplified graphical representation of the Markov model showing only the treatment categories, rather than all the individual Markov states is represented in figure 1. Patients continuously enter the model (inflow) at the start of the cycle and can start on Hemodialysis, Peritoneal dialysis, or Transplantation. From there they can move between these treatment modalities. Diabetics and non-diabetics are modeled separately since the transition probabilities between the treatment modalities differ between these groups. Since the incidence of kidney failure is increasing, we will also model this in the cost-effectiveness analysis. This will be calculated using data from the database of *Nefrovisie*, a large national database with records of ESRD patients. As stated before, the model will include probabilistic sensitivity analysis using Monte Carlo simulation. This means that not only mean numbers of patients per year per treatment modality will be modeled, but also the uncertainty surrounding those mean number of patients, (i.e. 95% confidence intervals). Transition probabilities and incidence rates will be based on primary data and will include uncertainty. Costs and utilities will be included in the model as distributions rather than point estimates.

Markov States: The Markov states are based on the treatments currently available in The Netherlands. These are: full care centre hemodialysis, limited care centre hemodialysis, home hemodialysis, continuous ambulatory peritoneal dialysis, continuous cyclic peritoneal dialysis, deceased donor kidney transplantation, and living donor kidney transplantation. Since transition

For peer review only - http://bmjopen.bmj.com/site/about/guidelines.xhtml

For peer review only

probabilities and costs may differ over time, i.e. a patient who is in his second year of hemodialysis has a different mortality chance than a patient who just started with hemodialysis, we will define separate Markov states for the 1st year of treatment and subsequent years of treatment for each specific modality. Incident patients that enter the model and prevalent patients that switch between treatment modalities are assigned to the 1st year Markov states, whereas patients that spend more than one year in any health state are transferred to the subsequent years of that same treatment modality.

Outcomes: The outcome of the implementation will be compared with the baseline situation; the situation before this programme was implemented. The effect size, in terms of an increase in LDKT, will be used from the RCT conducted earlier. Through sensitivity analyses an estimation can be made of the cost-effectiveness of the intervention whether this assumption is either an under- or overestimation. A critical assumption will be the extrapolation of the effects after the 2 years.

Quality Assurance

This study has also implemented a central quality assurance system. We hypothesize that the effectiveness is moderated by the protocol adherence of the team of healthcare professionals. In the implementation study, it might be possible that there are differences in the way teams and centers adhere to the protocol. Protocol adherence measures will be assessed by a third party by means of a telephone interview with the patients and the invitees. Patients and invitees are asked for their opinion and level of satisfaction regarding the way in which the intervention was delivered. Patients and invitees are asked to answer the 15-item TAM-scoring list. Items are rated on a Likert-scale (1 not at all – 5 very much). Only items that are rated with a 5, will be regarded as fully adherent. Items scored with a 1-4 will be regarded as non-adherent. The outcome of the protocol adherence will be associated with the gain in knowledge and communication skills. We will also look if there is a correlation between the protocol adherence and the amount of LDKTs. A part of the quality assurance is a training that all professionals who conduct the home interventions must take part in. During this training, issues are discussed such as: how to convey uniform and complete information to the patient, how to behave during the home visits, how to create an alignment of the goals of the home visit with the patients, how to assist the patient in inviting friends and relatives, how to deal with emotional moments, how to

For peer review only - http://bmjopen.bmj.com/site/about/guidelines.xhtml

discuss delicate topics with respect to individual feelings and opinions and finally, how to ensure no detrimental psychosocial effects of the education occur for the patients and his/her family/friends. All these aspects can be executed in different ways by the educators. A supervisor evaluates the home visits with each team separately every six weeks and discuss difficult cases. After these meetings the supervisor is graded by the educators through a 10-item questionnaire regarding the content of the teaching and the interpersonal delivery of the supervisor. Furthermore, the supervisor will bring together all educators for a so-called intervision meeting every three months. These intervision sessions are meant to discuss the home visits with each other in order to learn from each other and to keep the procedures similar.

Discussion

We presented a protocol for assessing the cost-effectiveness of our home-based educational programme and its generalizability. The implementation of the educational programme might both benefit patients and society.

Cost-effectiveness: If indeed our hypotheses are confirmed, and the home-based educational programme is cost-effective, then there are convincing arguments to make the programme standard care in The Netherlands. Health insurers already expressed their interest in the programme; this implementation study is supported by *Zorgverzekeraars Nederland (*Health insurers The Netherlands*)*, which is the 'umbrella organization' of all health insurers in The Netherlands. Additionally, the Dutch Kidney Foundation supports the programme and contributed through three grants in the developmental phases of the home-based educational programme. The Dutch Kidney Foundation is a non-profit organization which subsidizes research and innovation in nephrology and renal transplantation care. Indeed, the health insurance companies have good reason to be interested, as dialysis is costly. In The Netherlands, 1% of the total healthcare budget is spent on ESRD patients, who only constitute 0.0006% of the population [26, 27]. Furthermore, transplantation is associated with higher quality of life for ESRD patients compared to dialysis treatment. It is therefore valuable, from both patient and societal perspective, to conduct a complete and extensive cost-effective analysis and consequently to follow up those results in terms of policy.

For peer review only - http://bmjopen.bmj.com/site/about/guidelines.xhtml

Quality Assurance System: Protocol adherence may be of importance to guarantee the effects of the home-based education. First, we expect a positive relation of adherence with outcome in terms of communication, knowledge and the number of transplantations. Second, any problems or regrets of donors and/or patients can only be justified if the evidence based protocol was followed. The protocol has also been developed after thorough ethical consideration (23), which justified all characteristics of the programme. It can therefore be argued that health insurance make reimbursement indispensable of the degree of protocol adherence of healthcare suppliers. Moreover, they should facilitate the quality assurance system as an integral part of the programme and ensure that the quality of the interventions is independent of the healthcare suppliers.

*Limitations:*Investigating the (cost-)effectiveness of the home-based educational programme has its limitations. In health economic modelling, there is always a trade-off between the feasibility and transparency of the cost-effectiveness model and the level of details of real life conditions as represented in the model. The more details, the more the model resembles real life, but the down side is that data should be available at that same level of detail and that the model becomes too complex in its feasibility. An example is that we assume that the mortality on dialysis is the same in the second and following years on dialysis. Hence, we know from literature that the mortality chance changes, but the data in later years is scarce and again the model would become more complex, as more 'tunnels states' have to be introduced. We expect that this trade-off will be most prominent in the transition changes between health states. We expect less obvious trade-offs for costs and utility assessment, as we have sufficient data for those variables.

Cooperation. This investigation is a cooperation between many parties, who all have expressed their support. Obviously, it is possible that this support can be withdrawn for several reasons. For instance, we depend on data from a large national database with records of patient with ESRD (*Nefrovisie*). Hence, much efforts are and will be put in preserving relationships and communication in order to maximize fair successful implementation chances for the programme.

Ethical considerations. Another challenge that we face, is the ethical consideration of promoting living kidney donation through a home-based intervention. Previous studies on the ethics of this argued that such promotion is justified, only when the conditions are met, such as 1) participation must be completely voluntary throughout the intervention, 2) no undue pressure

For peer review only - http://bmjopen.bmj.com/site/about/guidelines.xhtml

should be put on the participants, 3) the education is neutral and non-directive and 4) the purpose and the procedure should be clear to all participants [28, 29]. That does not mean there are no negative consequence whatsoever, but it does mean that the positive outcomes outweigh the negative. It could well be that a patient and/or a donor may regret the decision to have undertaken a transplantation with a kidney of a living donor, and that the donor and/or patient, in hindsight, may have felt undue pressure to donate a kidney. Adherence to the protocol will minimize these potential negative effects. However, it is possible that regret or pressure could lead to negative publicity for the programme. That is a risk since such negative publicity could impede the chances of implementing the programme as standard care [30]. Especially considering that deceased organ donation is currently subject to controversy in The Netherlands since the government and parliament have accepted a donor law. This law is an opt-out system entailing a positive 'no-objection' deceased donor organ donor registration as a default for all Dutch citizens [31]. Given this potential harm due to negative publicity it is crucial to have 1) a protocol which is justifiable from a medical ethical perspective, 2) widespread support from the various organizations involved and 3) a high quality in terms of protocol adherence and trained adequate educators. If these conditions are met, the quality of the process then justify its outcome, which is a subtle trade-off between positive and negative outcomes.

Implementation. Another challenge is the generalizability of the results of the previous effect study on home-based interventions done in Rotterdam. The randomized controlled trial in the Rotterdam transplant area has shown that the home-based educational programme leads up to four times more LDKTs. The trial took place at the academic transplant center (Erasmus Medical Center in Rotterdam) where extensive efforts were already undertaken as part of the standard care to promote LDKTs [3, 32]. It is possible that in other transplant areas in The Netherlands, with less experience regarding promoting LDKT, effectiveness in terms of amount of LDKTs may differ. On one hand this more cautious attitude may lead to lower results than in the Rotterdam transplant area. Hence, organizational conditions within those transplant centers may not optimally facilitate the favorable results of the interventions. On the other hand, if the number of LDKT was lower than in the Rotterdam area, and the uptake of the intervention is high, the effect could even be higher than in the Rotterdam area. This is due to a higher effect potential in those centers where living donation was not promoted as actively.

For peer review only - http://bmjopen.bmj.com/site/about/guidelines.xhtml

Learning curve. As with all new programmes, educators will inherently experience a learning curve during the first part of the implementation, which could influence the effectiveness of the programme. For instance, the goal of 50 patients per year per academic transplant center and the goal of 20 patients per regional hospital may not be reached. Regular supervision, (on the job) training and peer-to-peer coaching may help to overcome this, but a learning curve is unavoidable.

Policy implications: One of the main pillars of an efficient health care system is the ability to provide effective care to patients when needed. It is therefore necessary to have information on the effectiveness of the interventions and their cost to convince policy makers to reimburse the treatment. If the analysis confirms the effectiveness as well as the cost-effectiveness of the home-based educational programme, we recommend that this intervention should be part of standard-care.

If the home-based educational interventions would become standard-care, this could have several implications. First, it can be expected that patients who are unable to find a living donor will nevertheless profit from an increase number of living kidney donations, as the demand for deceased donor kidneys drops. In other words, the increase in living donation will further lower the waiting list for deceased donor donation as well and thereby increase the chance of a deceased donor donation for those patients without a living donor increases.

Second, the composition of the population of patients with ESRD may change. For instance, it can be expected that the proportion of patients on dialysis will drop and patients with a life sustaining transplanted kidney will increase. This might have an influence on the demand for dialysis centers and the need for transplantation facilities. We, therefore, aim to incorporate this in a so-called dynamic model to estimate the prevalence over time. When modelling the prevalence, we could make estimates of the need for dialysis centers and transplantations facilities. However, modelling the facility is surrounded by uncertainty. For instance, dialysis centers may have financial incentives to fulfill dialysis capacity. If this would be the case, there will be no monetary benefits for society by increasing the transplants facilities if the proportion of dialysis capacity remains the same. This would mean that there will only be an increase in the average quality of life of ESRD-patients.

Finally, a cost-effectiveness analysis only might not be sufficient to set policy change in motion. Therefore, we anticipate that a policy recommendation accompanied with a BIA will

For peer review only - http://bmjopen.bmj.com/site/about/guidelines.xhtml

also be required. A BIA addresses the expected changes in the expenditure of a health care system after the adoption of a new intervention. It can also be used for budget and resource planning [33, 34].

Conclusion: If our hypotheses are confirmed, we hope by presenting an extensive cost-effectiveness analysis, a BIA, and a policy recommendation that policy change will be set in motion, which again would benefit both ESRD-patients and society.

For peer review only

Declarations

Abbreviations

BIA: Budget Impact Analysis; ESRD: End-Stage Renal Disease; LDKT: Living Donor Kidney Transplantation; RCT: Randomized Controlled Trial; RRT: Renal Replacement Therapies; QALY: Quality Adjusted Life Years; TAM; Treatment Adherence Measures.

Acknowledgements

All authors express their gratitude to the participating hospitals, the educators and their supervisors.

Funding

The research is funded by the Dutch Kidney Foundation (Nierstichting). The implementation is supported by Dutch Health Insurers (Zorgverzekeraars Nederland).

Availability of data and materials

Not applicable.

Author contributions

SR drafted the manuscript. MO, MV, JB, WW, EM, SI provided significant critical intellectual contributions. All authors reviewed and approved the final version of this manuscript and agrees to be accountable for all aspects of this work.

Consent for publication

Not applicable.

Competing interests

All authors have no competing interests to report.

For peer review only - http://bmjopen.bmj.com/site/about/guidelines.xhtml

Ethics and dissemination

Ethical approval is obtained through all participating hospitals. Results will be dissemnitated through peer-reviewed publications and scientific presentations. Results of the cost-effectiveness of the educational programme will also be disseminated to the Dutch National Health Care Institute.

For peer review only

References

1. Mahillo B, Carmona M, Alvarez M, White S, Noel L, Matesanz R: **2009 global data in organ donation and transplantation: activities, laws, and organization**. *Transplantation* 2011, **92**(10):1069-1074.
2. Kranenburg LW, Zuidema WC, Weimar W, Hilhorst MT, IJzermans JN, Passchier J, Busschbach JJ: **Psychological barriers for living kidney donation: how to inform the potential donors**. *Transplantation* 2007, **84**:965-971.
3. Ismail SY, Luchtenburg AE, Timman R, Zuidema WC, Boonstra C, Weimar W, Busschbach JJ, Massey EK: **Home-based family intervention increases**

For peer review only - http://bmjopen.bmj.com/site/about/guidelines.xhtml

knowledge, communication and living donation rates: a randomized controlled trial. *Am J Transplant* 2014, **14**(8).
4. Massey EK, Gregoor PJ, Nette RW, van den Dorpel MA, van Kooij A, Zietse R, Zuidema WC, Timman R, Busschbach JJ, Weimar W: **Early home-based group education to support informed decision-making among patients with end-stage renal disease: a multi-centre randomized controlled trial**. *Nephrol Dial Transplant* 2016, **31**(5):823-830.
5. Rodrigue JR, Cornell DL, Lin JK, Kaplan B, Howard RJ: **Increasing live donor kidney transplantation: a randomized controlled trial of a home-based educational intervention**. *Am J Transplant* 2007, **7**(2):394-401.
6. Henggeler SW, Schoenwald SK, Borduin CM, Rowland MD, Cunningham PB: **Multisystemic Therapy for Antisocial Behavior in Children and Adolescents**. New York: The Guilford Press; 2009.
7. de Wit GA, H. RP, de Charro FT: **Eonomic evaluation of end stage renal disease treatment**. *Health policy* 1998, **44**(3):215-232.
8. Wyld M, Morton RL, Hayen A, Howard K, Webster AC: **A systematic review and meta-analysis of utility-based quality of life in chronic kidney disease treatments**. *PLoS Med* 2012, **9**(9):e1001307.
9. Liem YS, Bosch JL, Hunink MG: **Preference-based quality of life of patients on renal replacement therapy: a systematic review and meta-analysis**. *Value Health* 2008, **11**(4):733-741.
10. Hartmann A: **Managing a living donor program: the Oslo experience**. Presented at the 11th Conference of the European Society of Organ Transplantation*.* Venice. Septemeber 20-24, 2003
11. Ismail SY, Claassens L, Luchtenburg AE, Roodnat JI, Zuidema WC, Weimar W, Busschbach JJ, Massey EK: **Living donor kidney transplantation among ethnic minorities in the Netherlands: a model for breaking the hurdles**. *Patient Educ Couns* 2013, **90**(1):118-124.
12. Ismail SY, Luchtenburg AE, Zuidema WC, Boonstra C, Weimar W, Massey EK, Busschbach JJ: **Multisystemic engagement & nephrology based educational intervention: a randomized controlled trial protocol on the kidney team at home-study**. *BMC Nephrology* 2012, **13**:62-70.
13. Ismail SY, Timmerman L, Timman R, Luchtenburg AE, Smak Gregoor PJH, Nette RW, van den Dorpel MA, Zuidema WC, Weimar W, Massey EK *et al*: **A psychometric analysis of Rotterdam Renal Replacement Knowledge-Test (R3K-T) using item response theory**. *Transplant International* 2013, **26**:1164-1172.
14. Chapman JE, Sheidow AJ, Henggeler SW, Halliday-Boykins C, Cunningham PB: **Developing a Measure of Therapist Adherence to Contingency Management: An Application of the Many-Facet Rasch Model**. *J Child Adolesc Subst Abuse* 2008, **17**(3):47-68.

For peer review only - http://bmjopen.bmj.com/site/about/guidelines.xhtml

15. Drummond MF, Sculpher MJ, Claxton K, Stoddart GL, Torrance GW: **Methods for the Economic Evaluation of Health Care Programmes**. Oxford: Oxford University; 2015.
16. Zorginstituut Nederland: **Richtlijn voor het uitvoeren van economische evaluaties in de gezondheidszorg**. In. Diemen: Nederland Zorginstituut 2015.
17. Kuiper D, Brouwer S, Groothogg J, van der Mei S: **Arbeidsparticipatie na niertransplantatie: een prospectieve studie**. In. Groningen: Rijksuniversiteit Groningen; 2009.
18. Jarl J, Gerdtham UG, Desatnik P, Prutz KG: **Effects of Kidney Transplantation on Labor Market Outcomes in Sweden**. *Transplantation* 2018, **102**(8):1375-1381.
19. Hoang VL, Green T, Bonner A: **Informal caregivers' experiences of caring for people receiving dialysis: A mixed-methods systematic review**. *J Ren Care* 2018, **44**(2):82-95.
20. Harris A, Cooper BA, Li JJ, Bulfone L, Branley P, Collins JF, Craig JC, Fraenkel MB, Johnson DW, Kesselhut J *et al*: **Cost-effectiveness of initiating dialysis early: a randomized controlled trial**. *Am J Kidney Dis* 2011, **57**(5):707-715.
21. Wu AW, Fink NE, Marsh-Manzi JV, Meyer KB, Finkelstein FO, Chapman MM, Powe NR: **Changes in quality of life during hemodialysis and peritoneal dialysis treatment: generic and disease specific measures**. *J Am Soc Nephrol* 2004, **15**(3):743-753.
22. Doubilet P, Begg CB, Weinstein MC, Braun P, J. MB: **Probabilistic Sensitivity Analysis Using Monte Carlo Simulation**. *Med Decis Making* 1985, **5**(2):157-177.
23. Briggs AH, Goeree R, Blackhouse G, O'Brien BJ: **Probabilistic analysis of cost-effectiveness models: choosing between treatment strategies for gastroesophage reflux disease**. *Med Decis Making* 2002, **22**:290-308.
24. Briggs AH: **Handling uncertainty in cost-effectiveness models**. *Pharmacoeconomics* 2000, **17**(5):479-500.
25. Sonnenberg FA, Beck JR: **Markov Models in Medical Decision Making: A Pratical Guide**. *Med Decis Making* 1993, **13**:322-338.
26. **Ranglijst ziekten op basis van zorgkosten** [https://www.volksgezondheidenzorg.info/ranglijst/ranglijst-ziekten-op-basis-van-zorgkosten]
27. **Feiten en cijfers over behandeling nierfalen** [https://www.nierstichting.nl/leven-met-een-nierziekte/feiten-en-cijfers/]
28. Hilhorst MT, Kranenburg LW, Busschbach JJ: **Should health care professionals encourage living kidney donation**. *Medicine, Health Care and Philosophy* 2006, **10**:81-90.
29. Massey EK, Hilhorst MT, Nette RW, Smak Gregoor PJH, van den Dorpel MA, van Kooij A, Zuidema WC, Zietse R, Busschbach JJ, Weimar W: **Justification**

For peer review only - http://bmjopen.bmj.com/site/about/guidelines.xhtml

for a home-based education programme for kidney patients and their social network prior to initiation of renal replacement therapy. *J Med Ethics* 2011, **37**:677-681.
30. Birkland TA: **Focusing Events, Mobilization, and Agenda Setting**. *Journal of Public Policy* 1998, **18**(1):53-74.
31. Eleftheriou-Smith L: **Organ Donation set to become automatic for Dutch people unless they opt out**. In: *Independent.* 2016.
32. Roodnat JI, Kal-V Gestel JA, Zuidema WC, van Noord MAA, van de Wetering J, IJzermans JN, Weimar W: **Successful Expansion of the Living Donor Pool by Alternative Living Donation Programs**. *Am J Transplant* 2009, **9**(9):2150-2156.
33. Niezen MG, de Bont A, Busschbach JJ, Cohen JP, Stolk EA: **Finding legitimacy for the role of budget impact in drug reimbursement decisions**. *Int J Technol Assess Health Care* 2009, **25**(1):49-55.
34. Sullivan SD, Mauskopf JA, Augustovski F, Caro JJ, Lee KM, Minchin M, Orlewska E, Penna P, Rodriguez Barrios J-M, Wen-Yi S: **Budget Impact Analysis - Principles of Good Practice: Report of the ISPOR 2012 Buget Impact Analysis Good Practice II Task Force**. *Value in Health* 2014, **17**:5-14.

Figure legends

Figure 1: A simplified graphic representation of the Markov Model with the different health states and the transition possibilities between the health states.

Table 1: overview of parameters and sources in the Markov Model.

For peer review only

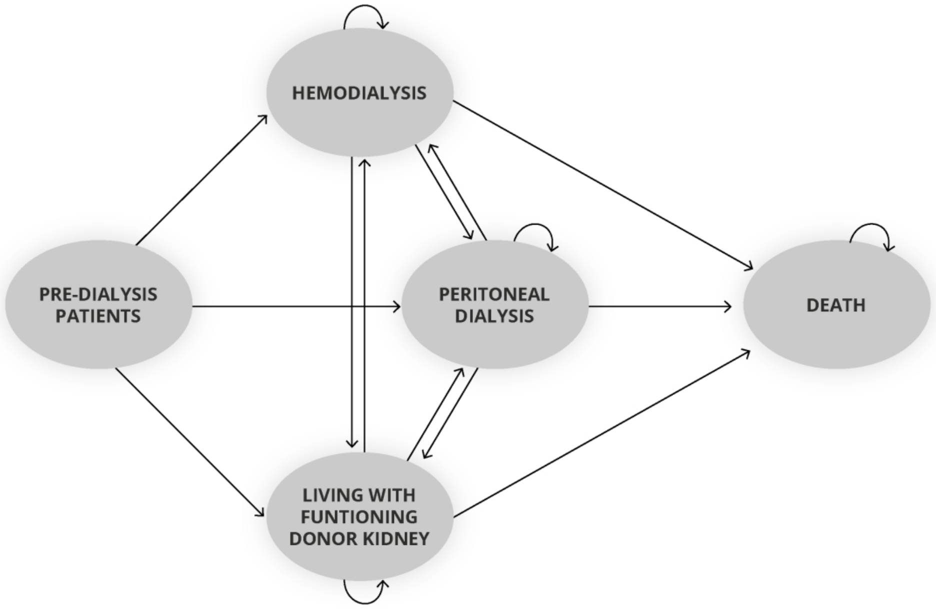

A simplified graphic representation of the Markov Model with the different health states and the transition possibilities between the health states.

234x151mm (300 x 300 DPI)
